# β-aminoisobutyric acid attenuates LPS-induced inflammation and insulin resistance in adipocytes through AMPK-mediated pathway

**DOI:** 10.1186/s12929-018-0431-7

**Published:** 2018-03-28

**Authors:** Tae Woo Jung, Hyung Sub Park, Geum Hee Choi, Daehwan Kim, Taeseung Lee

**Affiliations:** 1 Research Administration Team, Seoul National University Bundang Hospital, 166 Gumi-ro, Bundang-gu, Seongnam, 463-707 Korea; 2 Department of Surgery, Seoul National University Bundang Hospital, Seoul National University College of Medicine, 166 Gumi-ro, Bundang-gu, Seongnam, 463-707 Korea; 3 Department of Surgery, Seoul National University College of Medicine, Seoul, Korea

**Keywords:** BAIBA, AMPK, NFκB, Inflammation, Insulin resistance, Adipocyte

## Abstract

**Background:**

β-aminoisobutyric acid (BAIBA) is produced in skeletal muscle during exercise and has beneficial effects on obesity-related metabolic disorders such as diabetes and non-alcoholic fatty liver disease. Thus, it is supposed to prevent high fat diet (HFD)-induced inflammation and insulin resistance in adipose tissue though anti-inflammatory effects in obesity. Previous reports have also demonstrated strong anti-inflammatory effects of BAIBA.

**Methods:**

We used BAIBA treated fully differentiated 3T3T-L1 mouse adipocytes to investigate the effects of exogenous BAIBA on inflammation and insulin signaling in adipocytes. Insulin signaling-mediated proteins and inflammation markers were measured by Western blot analysis. Secretion of pro-inflammatory cytokines were measured by ELISA. Lipid accumulation in differentiated 3 T3-L1 cells was stained by Oil red-O. Statistical analysis was performed by ANOVA and student’s *t* test.

**Results:**

BAIBA treatment suppressed adipogenesis assessed by adipogenic markers as well as lipid accumulation after full differentiation. We showed that BAIBA treatment stimulated AMP-activated protein kinase (AMPK) phosphorylation in a dose-dependent manner and lipopolysaccharide (LPS)-induced secretion of pro-inflammatory cytokines such as TNFα and MCP-1 was abrogated in BAIBA-treated 3 T3-L1 cells. Treatment of 3 T3-L1 cells with BAIBA reduced LPS-induced NFκB and IκB phosphorylation. Furthermore, BAIBA treatment ameliorated LPS-induced impairment of insulin signaling measured by IRS-1 and Akt phosphorylation and fatty acid oxidation. Suppression of AMPK by small interfering (si) RNA significantly restored these changes.

**Conclusions:**

We demonstrated anti-inflammatory and anti-insulin resistance effects of BAIBA in differentiated 3 T3-L1 cells treated with LPS through AMPK-dependent signaling. These results provide evidence for the beneficial effects of BAIBA not only in liver and skeletal muscle cells but also in adipose tissue.

**Electronic supplementary material:**

The online version of this article (10.1186/s12929-018-0431-7) contains supplementary material, which is available to authorized users.

## Background

Low-grade chronic adipose tissue inflammation and macrophage infiltration into adipose tissue are main characteristics of adipose tissue dysfunction in obesity [1, 2]. Abnormal secretion of pro-inflammatory cytokines by adipose tissue and macrophage infiltration results in the development of metabolic disorders such as insulin resistance and atherosclerosis [3].

Although regular exercise has beneficial effects on atherosclerosis in humans [4], the underlying mechanisms remain unclear. β-aminoisobutyric acid (BAIBA) is a natural catabolite of thymine that has been shown to attenuate obesity via stimulation of fatty acid oxidation and suppression of lipogenesis in animal models [5]. Recently, BAIBA was identified as a myokine released by skeletal muscle through a proliferator-activated receptor-gamma coactivator-1α (PGC-1α)-dependent pathway during physical activity. BAIBA stimulates the browning of white adipose tissue and fatty acid oxidation in the liver via a PPAR α-mediated pathway [6].

AMPK is an energy sensor that maintains cellular energy homeostasis [7] and inhibits the nuclear factor-κB (NFκB)-dependent inflammatory process [8]. AMPK activation by 5-aminoimidazole-4-carboximide ribonucleotide (AICAR) increases insulin sensitivity in various organs such as skeletal muscle [9], liver [10], and adipose tissue [11]. Jung et al. reported that BAIBA attenuated inflammation and insulin resistance in high fat diet (HFD)-fed mice, and that this effect was negated by siRNA-mediated suppression of AMPK [12]. BAIBA-mediated AMPK signaling has been reported to attenuate ER stress in response to hyperlipidemia, thereby ameliorating hepatic apoptosis [13]. Furthermore, BAIBA was shown to ameliorate renal fibrosis through suppression of a reactive oxygen species-mediated pathway [14]. Therefore, BAIBA-induced AMPK seems to play an important role in the pathogenesis of metabolic disorders.

Here, we investigated [5] the effect of BAIBA on LPS-induced inflammation and insulin resistance in differentiated 3 T3-L1 cells; [1] the mechanisms of BAIBA-mediated suppression of inflammation and insulin resistance through AMPK-mediated pathway.

## Methods

### Cell cultures, reagents, and antibodies

The mouse pre-adipocytes 3 T3-L1 (ATCC, Manassas, VA, USA) were cultured in Dulbecco’s modified eagle medium (DMEM; Invitrogen, Carlsbad, CA, USA) supplemented with 10% fetal bovine serum (Invitrogen), 100 units/mL penicillin, and 100 μg/mL streptomycin (Invitrogen). Cells were cultured in a humidified atmosphere of 5% CO_2_ at 37 °C. Differentiation was induced 48 h post confluence (day 2) by cultivation in culture medium supplemented with 1 μM insulin, 0.5 mM IBMX and 0.5 μg/ml dexamethansone for 2 days. After another 2 days in medium containing 1 μM insulin. Human primary adipocytes (Zenbio, Research Triangle Park, NC, USA) were fed with Omental Adipocyte Medium (Zenbio). Primary adipocytes were cultured in a humidified atmosphere containing 5% CO_2_ at 37 °C pending uses. Differentiated 3 T3-L1 cells or human primary adipocytes were treated with 10 μg/ml LPS (Sigma, St Louis, MO, USA) and 0–30 μM BAIBA (Sigma) for 10 days. Differentiated 3 T3-L1 cells or human primary adipocytes were treated with 0–5 μM glimepiride (Sigma) [15] and 0–10 mM metformin (Sigma) [16] for 24 h. Human primary adipocytes were treated with 10 μM compound C (Sigma) for 24 h. Insulin (10 nM) was used to stimulate insulin signaling (insulin receptor substrate (IRS-1) and Akt) for 3 min. Anti-phospho Akt (Ser473; 1:1000), anti-Akt (1:1000), anti-phospho AMPK (Thr172; 1:1000), anti-AMPK (1:2500), anti-phospho NFκBp65 (1:1000), anti- NFκBp65 (1:2500), anti-phospho IκB (1:1000), anti-IκB (1:1000), anti-phospho ACC (1:1000), anti-ACC (1:1000), and anti-adiponectin antibodies were purchased from Cell Signaling Technology (Beverly, MA, USA). Anti-CPT1 (1:2000) and anti-β-actin (1:5000) were obtained from Santa Cruz Biotechnology (Santa Cruz, CA, USA).

### Western blot analysis

Differentiated 3 T3-L1 cells were harvested and proteins were extracted with lysis buffer (PRO-PREP; Intron Biotechnology, Seoul, Republic of Korea) for 60 min at 4 °C. Protein samples (30 μg) were subjected to 12% SDS-PAGE, transferred to a nitrocellulose membrane (Amersham Bioscience, Westborough, MA, USA), and probed with the indicated primary antibodies followed by secondary antibodies conjugated with horseradish peroxidase (Santa Cruz Biotechnology). The signals were detected using enhanced chemiluminescence (ECL) kits (Amersham Bioscience).

### RNA extraction and quantitative real-time PCR

Total RNAs from harvested hepatocytes were isolated using TRIzol reagent (Invitrogen, Carlsbad, CA). Gene expression was measured by quantitative real-time PCR (qPCR) using the fluorescent TaqMan 5’nuclease assay on an Applied Biosystems 7000 sequence detection system (Foster City, CA, USA). qPCR was performed using cDNA with 2× TaqMan Master Mix and the 20 × premade TaqMan gene expression assays (Applied Biosystems). qPCR conditions were 95 °C for 10 min, followed by 40 cycles of 95 °C for 15 s and 60 °C for 1 min. The PCR primer mix for mouse PPARγ (Mm00440940_m1), FABP4 (Mm00445878_m1), adiponectin (Mm00456425_m1), and fatty acid synthase (Mm00662319_m1) were used. The mRNA of β-actin was quantified as an endogenous control, using primers: 5’-CGATGCTCCC CGGGCTGTAT-3′ and 5’-TGGGGTACTTCAGGGTCAGG-3′.

### Transfection with siRNAs for gene silencing in cells

siRNA oligonucleotides (20 nM) specific for AMPKα 1/2 were purchased from Santa Cruz Biotechnology. To suppress gene expression, cell transfection was performed with Lipofectamine 2000 (Invitrogen) according to the manufacturer’s instructions. In brief, the cells were grown to 60% - 70% confluence, followed by serum starvation for 12 h after 3 T3-L1 cell differentiation. The cells were transfected with validated siRNA or scramble siRNA at a final concentration of 20 nM in the presence of transfection reagent. After transfection, the cells were harvested at 36 h for protein extraction and additional analysis.

### Enzyme linked immunosorbent assay (ELISA)

Mouse serum TNFα and MCP-1 were measured with each ELISA kit (R&D Systems, Minneapolis, MN, USA) following the manufacturer’s instructions.

### Measurement of glucose uptake and acetyl-CoA and ATP content

Glucose uptake levels were measured using Glucose Uptake Assay Kit™ (Abcam, Cambridge, MA, USA). Briefly, proliferating and differentiating 3 T3-L1 cells were seeded at 5 × 10^5^ cells/well in black walled / clear bottom 96-well plates (Corning, Inc., Corning, NY, USA) in DMEM containing 10% FBS. Upon reaching a confluency of 95%, differentiation was induced with differentiating media. After 48 h, media was changed to media containing 10 μg/ml LPS or 0–30 μM BAIBA for 10 days. Following treatment, media was removed from wells and treated with 10 nM insulin and 1 mM 2-Deoxyglucose (2-DG) for 30 min. Afterwards plates were centrifuged for 1 min at 58 g and incubated for 1 h at room temperature. After 2-DG taken up by the cells were extracted by extraction buffer in kit, 2-DG uptake levels were then measured at a wavelength of OD 412 nm on a BioTek Synergy HT plate reader (BioTek Instruments, Inc., Winooski, VT, USA). Intracellular levels of acetyl-CoA were measured using PicoProbe Acetyl CoA Assay Kit™ (Abcam) and intracellular ATP levels were measured using ATP Assay Kit™ (Abcam) in differentiated 3 T3-L1 cells were measured according to the manufacturer’s instructions.

### Long-chain acyl-CoA dehydrogenase (LCAD) expression in intact cells

LCAD expression was measured with Fatty Acid Oxidation Human In-Cell ELISA kit™ (Abcam) following the manufacturer’s directions.

### Cell viability assay

Cell viability was determined by 3-(4,5-dimethylthiazol-2-yl)-2,5-diphenyltetrazolium bromide (MTT) assay. In brief, MTT solution (Sigma) was added to experimental cell cultures in 96-well plates and incubated at 37 °C for 1 h. After washing five times with PBS, accumulated red formazan in the experimental cells was dissolved in dimethyl sulfoxide (DMSO) (Sigma). The optical density was used as an indicator of cell viability and was measured at 550 nm.

### Statistical analysis

Results are presented as the relative values (means ± SEM). All experiments were performed at least three times. Student’s *t* test or two-way ANOVA were used for statistical analysis. All analyses were performed using the SPSS/PC statistical program (version 12.0 for Windows; SPSS, Chicago, IL, USA).

## Results

### BAIBA suppresses lipid accumulation during differentiation of 3 T3-L1 cells

**Fig. 1:**
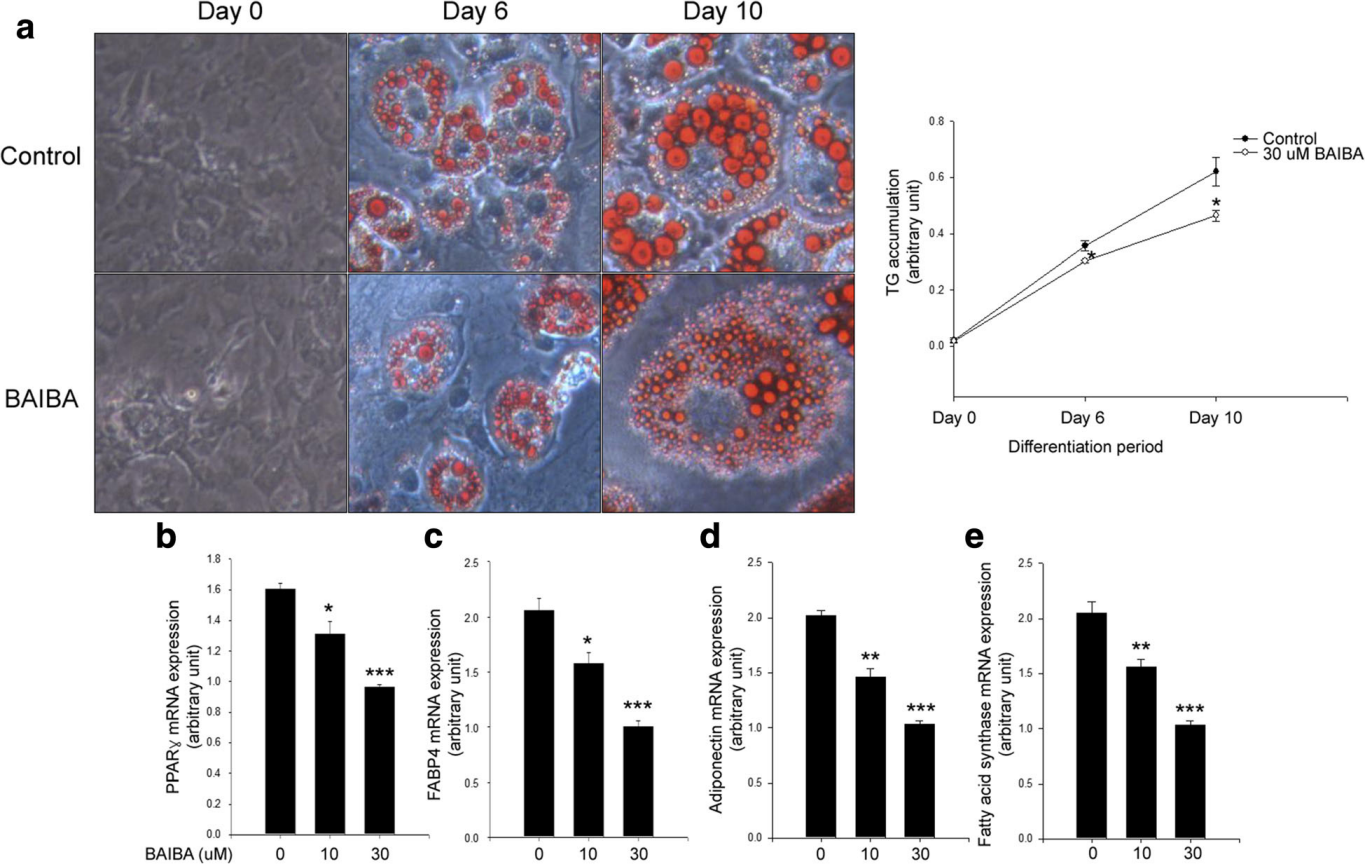
BAIBA suppresses TG accumulation in 3 T3-L1 cells during differentiation. **a** Oil-red O staining in differentiated 3 T3-L1 cells in the presence of BAIBA (30 μM) for 0, 6, or 10 days. TG accumulation was quantitated by modified TG assay kit. Quantitative real-time PCR analysis of PPARγ (**b**), FABP4 (**c**), adiponectin (**d**), and fatty acid synthase (**e**) mRNA expression in differentiated 3 T3-L1 cells treated with BAIBA (0–30 μM) for 10 days. Means ± SEM were obtained from three separated experiments. ^***^P < 0.001, ^**^P < 0.01, and ^*^P < 0.05 when compared to the control

BAIBA has been reported to suppress high fat diet-associated hepatic lipogenesis [13]. Therefore, we examined the effect of BAIBA on differentiation in 3 T3-L1 cells. As shown in Fig. 1, treatment of 3 T3-L1 cells with BAIBA significantly suppressed lipid accumulation (Fig. 1a) and lipogenesis-mediated PPARγ, FABP4, adiponectin, and fatty acid synthase mRNA expression (Fig. 1b-e).

### BAIBA ameliorates LPS-induced inflammation in differentiated 3 T3-L1 cells via AMPK-mediated pathway

**Fig. 2:**
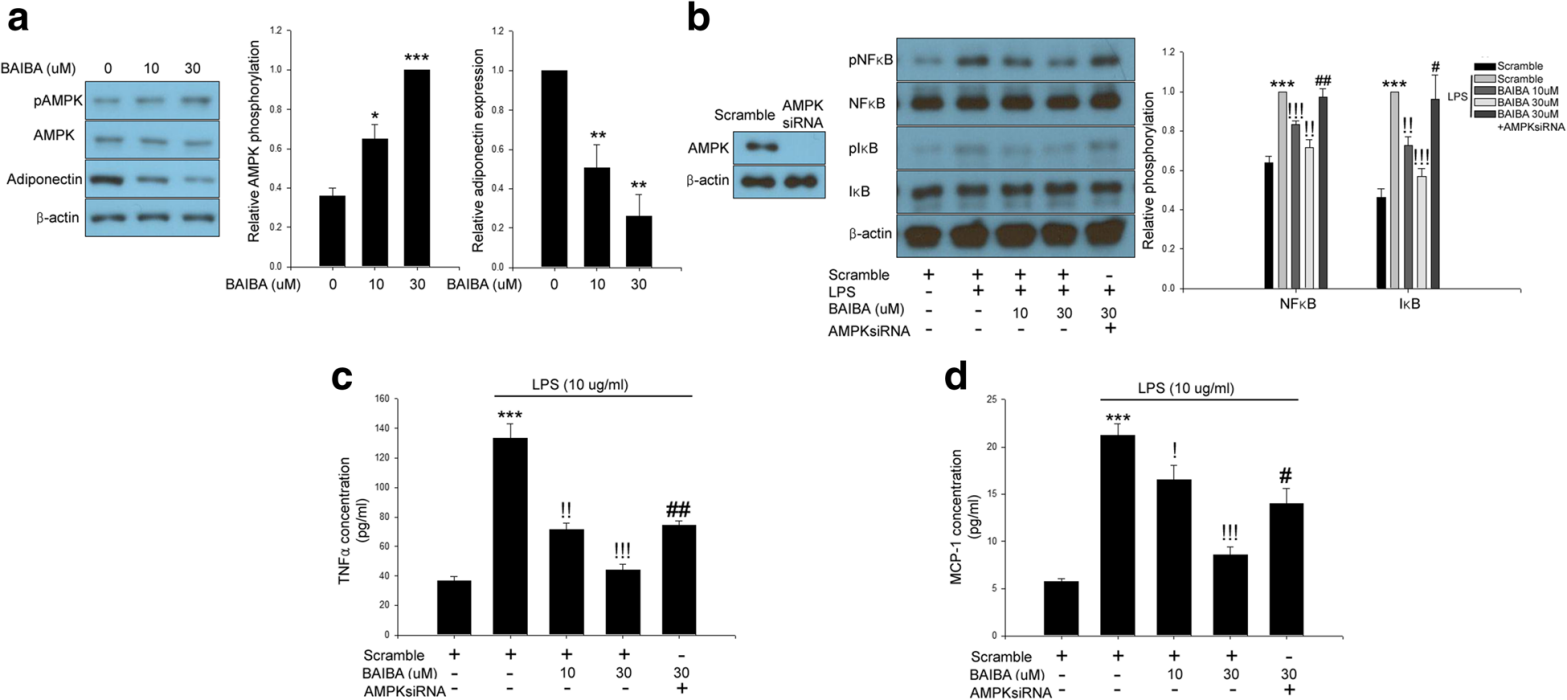
BAIBA ameliorates LPS-induced inflammation in differentiated 3 T3-L1 cells through AMPK-mediated pathway. **a** Western blot analysis of AMPK phosphorylation and adiponectin expression in differentiated 3 T3-L1 cells treated with BAIBA (0–30 μM) for 10 days. **b** Western blot analysis of LPS-induced NFκB and IκB phosphorylation in siRNA for AMPK transfected differentiated 3 T3-L1 cells treated with 10 μg/ml LPS and BAIBA (0–30 μM) for 10 days. Culture media analysis of (**c**) TNFα and (**d**) MCP-1 in siRNA for AMPK transfected differentiated 3 T3-L1 cells treated with 10 μg/ml LPS and BAIBA (0–30 μM) for 10 days. Means ± SEM were calculated data obtained from three independent experiments. ^***^P < 0.001, ^**^P < 0.01, and ^*^P < 0.05 when compared to the control.^!!!^*P* < 0.001,^!!^*P* < 0.01, and^!^*P* < 0.05 when compared to the LPS treatment. ^##^*P* < 0.01, and ^#^*P* < 0.05 when compared to the LPS plus BAIBA treatment

BAIBA prevented LPS-induced inflammatory signaling such as phosphorylation of NFκB and IκB and TNFα and MCP-1 secretion in differentiated 3 T3-L1 cells (Fig. 2). Since AMPK is a key target for the treatment of insulin resistance and type 2 diabetes [17] and AICAR ameliorates inflammatory responses [18], we next evaluated the effect of BAIBA on phosphorylation of AMPK in differentiated 3 T3-L1 cells. We found that BAIBA caused AMPK phosphorylation in a dose-dependent manner (Fig. 2a). Conversely, BAIBA inhibited adiponectin expression in differentiated 3 T3-L1 cells (Fig. 2a) similar with previous reports [19, 20]. These results suggest that BAIBA stimulates AMPK phosphorylation in an adiponectin-independent fashion. We next examined whether BAIBA-induced AMPK phosphorylation contributes to the suppression of LPS-induced inflammation in differentiated 3 T3-L1 cells. The suppressive effects of BAIBA on LPS-induced inflammation were significantly abrogated by siRNA for AMPK (Fig. 2b-d).

### BAIBA attenuates LPS-induced impairment of insulin signaling and insulin-stimulated glucose uptake in differentiated 3 T3-L1 cells via AMPK-dependent pathway

**Fig. 3:**
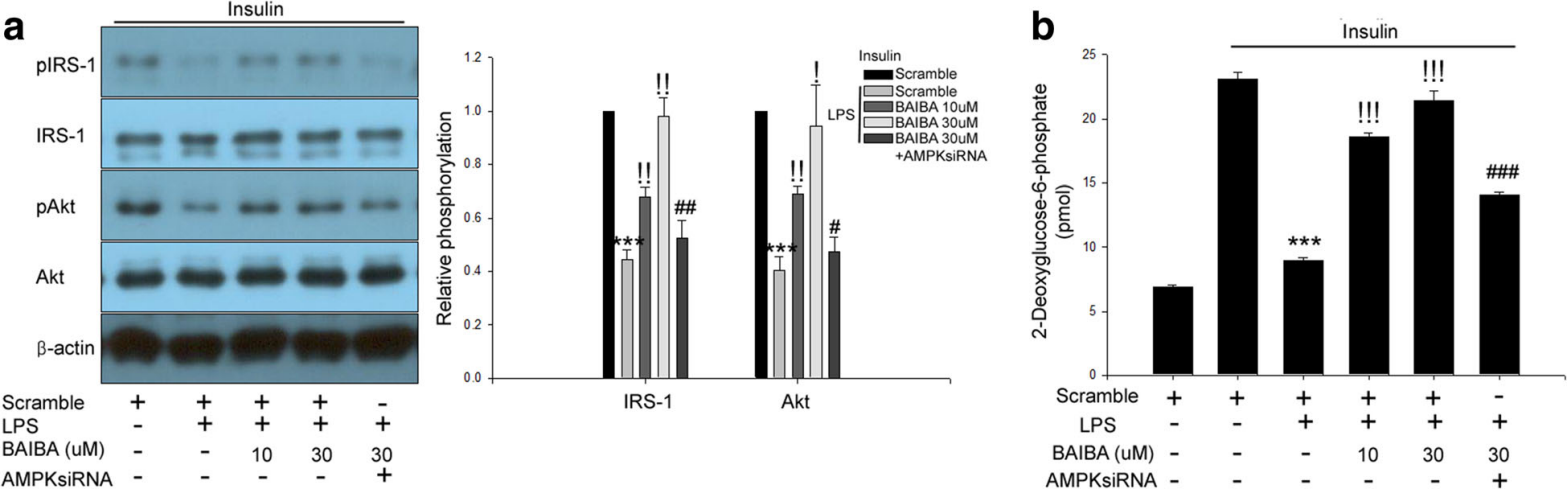
BAIBA attenuates LPS-induced insulin resistance in differentiated 3 T3-L1 cells through AMPK-mediated signaling. **a** Western blot analysis of phosphorylation of IRS-1 and Akt in siRNA for AMPK transfected differentiated 3 T3-L1 cells treated with 10 μg/ml LPS and BAIBA (0–30 μM) for 10 days. Human Insulin (10 nM) stimulates IRS-1 and Akt phosphorylation for 3 min. **b** 2-deoxyglucose uptake in scramble or AMPKsiRNA transfected differentiated 3 T3-L1 cells treated with 10 μg/ml LPS and BAIBA (0–30 μM) for 10 days. Human Insulin (10 nM) stimulates glucose uptake for 30 min. Means ± SEM were calculated data obtained from three independent experiments. ^***^P < 0.001 when compared to the insulin treatment.^!!!^*P* < 0.001,^!!^*P* < 0.01, and^!^*P* < 0.05 when compared to the insulin plus LPS treatment. ^###^*P* < 0.001, ^##^*P* < 0.01, and ^#^*P* < 0.05 when compared to the insulin, LPS plus BAIBA treatment

LPS treatment suppressed insulin-stimulated phosphorylation of IRS-1 and Akt, and glucose uptake in differentiated 3 T3-L1 cells. However, BAIBA blocked LPS-induced impairment of insulin signaling and insulin-stimulated glucose uptake (Fig. 3a and b). We also examined whether BAIBA-induced AMPK phosphorylation contributes to the suppression of LPS-induced insulin resistance in differentiated 3 T3-L1 cells. In Fig. 3a and b, siRNA for AMPK significantly abrogated the inhibitory effects of BAIBA on LPS-induced insulin resistance.

### BAIBA ameliorated LPS-induced impairment of fatty acid oxidation in differentiated 3 T3-L1 cells through AMPK signaling

**Fig. 4:**
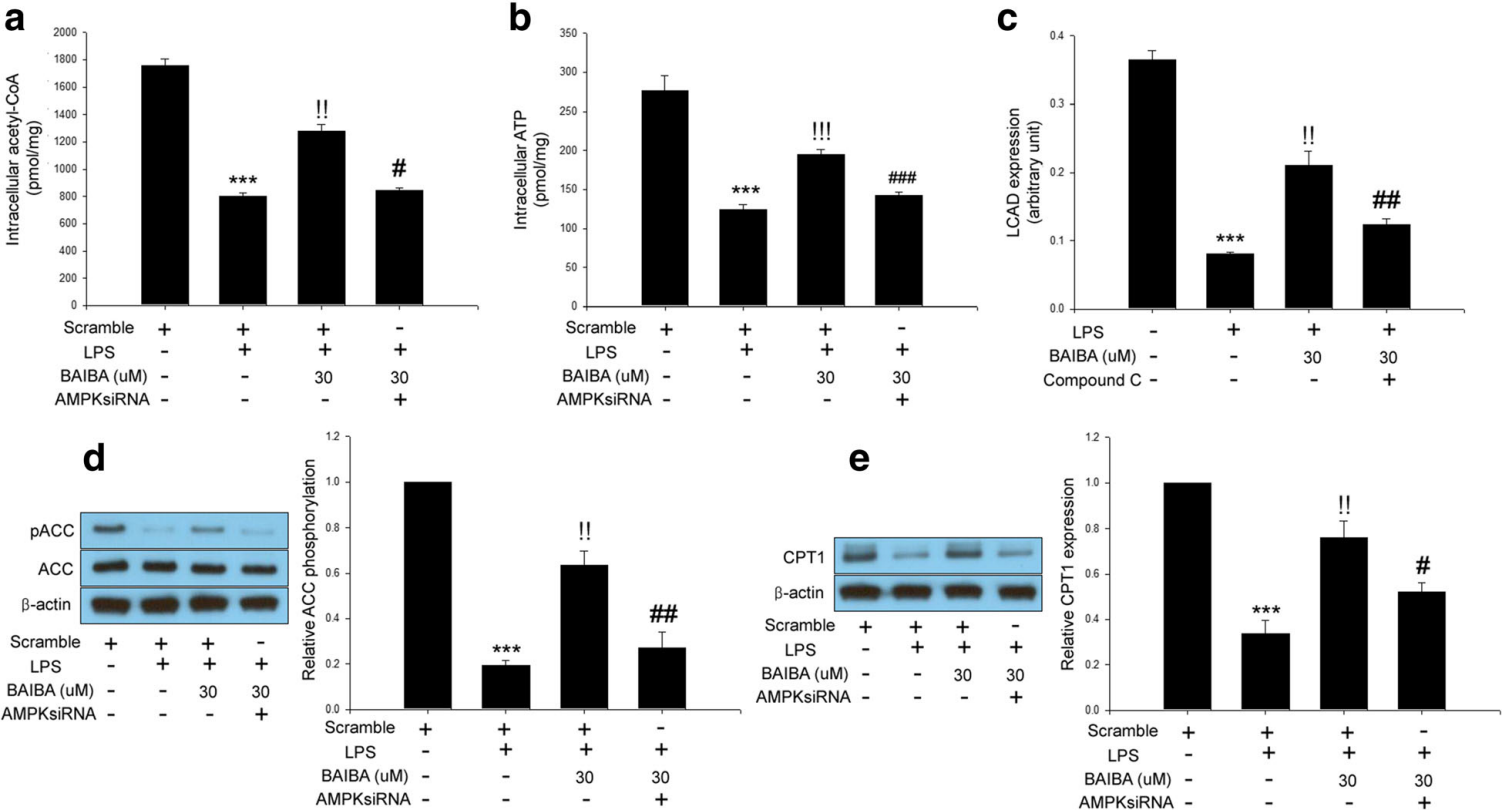
BAIBA stimulates fatty acid oxidation through AMPK signaling. Intracellular acetyl-CoA (**a**) and ATP levels (**b**) in scramble or AMPKsiRNA transfected differentiated 3 T3-L1 cells treated with 10 μg/ml LPS and BAIBA (0–30 μM) for 10 days. **c** LCAD expression levels in intact human primary adipocytes treated with 10 μg/ml LPS and BAIBA (0–30 μM) for 10 days and compound C (10 μM) for 24 h. Western blot analysis of ACC phosphorylation (**d**) and CPT1 expression (**e**) in differentiated 3 T3-L1 cells treated with 10 μg/ml LPS and BAIBA (0–30 μM) for 10 days. Means ± SEM were obtained from three separated experiments. ^***^P < 0.001 when compared to the insulin treatment.^!!!^*P* < 0.001,^!!^*P* < 0.01, and^!^*P* < 0.05 when compared to the insulin plus LPS treatment. ^###^*P* < 0.001, ^##^*P* < 0.01, and ^#^*P* < 0.05 when compared to the insulin, LPS plus BAIBA treatment

Koves et al. reported that mitochondrial overload and incomplete fatty acid oxidation cause insulin resistance in skeletal muscle [21]. Enhanced fatty acid oxidation improves insulin sensitivity in palmitate-treated adipocytes [22]. AMPK have been reported to augment fatty acid oxidation [23]. However, to the best of our knowledge, the effect of BAIBA on fatty acid oxidation in adipocytes has not been elucidated. Therefore, we next evaluated whether BAIBA-induced AMPK could induce fatty acid oxidation. To confirm the stimulation of fatty acid oxidation by BAIBA, we measured levels of acetyl-CoA and ATP, products of fatty acid oxidation, LCAD expression, ACC phosphorylation, and CPT1 expression. As expected, BAIBA treatment significantly elevated intracellular acetyl-CoA, ATP levels, ACC phosphorylation, and CPT1 expression in fully differentiated 3 T3-L1 cells. Furthermore, BAIBA treatment increased LCAD expression in intact human primary adipocytes. However, suppression of AMPK by siRNA or compound C, a specific AMPK inhibitor, restored these changes (Fig. 4).

### AMPK plays a critical role in BAIBA-mediated attenuation of inflammation and insulin resistance

To reaffirm importance of AMPK role in the beneficial effects of BAIBA, we examined the effects of glimepiride, an AMPK-independent drug, and metformin, an AMPK activator on inflammation and insulin resistance during same treatment time with BAIBA. Initially, we selected the treatment concentrations (1 μM glimepiride and 0.1 mM metformin for 10 days; 1 μM glimepiride and 10 mM metformin for 24 h) which do not affect cell viability and show maximal suppressive effects on inflammation (Additional file 1: Figure S1 and Additional file 2: Figure S2). BAIBA, metformin, and glimepiride ameliorated LPS-induced pro-inflammatory cytokines secretion and glucose uptake impairment. Notably, the effects of BAIBA and metformin on inflammation and insulin resistance are stronger than glimepiride for 10 days. Metformin and glimepiride treatment for 24 h showed similar results to results from 10 days treatment, whereas BAIBA had no effects on inflammation and insulin resistance (Additional file 3: Figure S3), suggesting that 24 h may be insufficient for BAIBA to work.

## Discussion

Obesity causes a low-grade chronic inflammatory state within adipose tissue accompanying elevated pro-inflammatory cytokine expression and infiltration of immune cells such as neutrophils and macrophages, further disseminating the inflammatory response and stimulating systemic inflammation [24, 25]. Adipose tissue and infiltrated macrophages secrete various pro-inflammatory cytokines that contribute to further immune cell infiltration leading to impaired hepatic and skeletal metabolic homeostasis and finally systemic insulin resistance [3, 26, 27]. Furthermore, Adipose tissue dysfunction caused by chronic inflammation leads to improper release and increase serum levels of free fatty acids (FFAs), which are known to contribute to insulin resistance through interfering insulin signaling [28]. The impairment of insulin signaling, leading to insulin resistance is a main characteristic of metabolic disorders and type 2 diabetes.

BAIBA has been previously reported to exert potent anti-inflammatory in hepatocytes [5] and skeletal muscle cells [12] and anti-diabetic effects in skeletal muscle cells [12] and hepatocytes [13]. In the current study, we investigated BAIBA effects on LPS-induced inflammation and insulin resistance in mouse adipocytes.

Herein, treatment of differentiated 3 T3-L1 cells with BAIBA led to the prevention of NFκB-mediated pro-inflammatory pathway activation and we also observed a significant decrease in pro-inflammatory cytokines TNFα and MCP-1 after LPS treatment. These results suggest that the anti-inflammatory effects of BAIBA in adipocytes are likely mediated by suppression of NFκB-mediated signaling and its downstream molecules.

The phosphatidylinositol 3-kinase (PI3K)/Akt pathway plays a central role in regulation of various intracellular signaling such as cell survival, proliferation, and differentiation [29]. This Akt activity is suppressed under obese conditions because of the phosphorylation of serine residues in IRS-1, resulting in impairment of the IRS-1-induced PI3K-mediated pathway [30] and inhibition of IRS-1-mediated Akt activation [31, 32]. We previously reported that BAIBA treatment reversed hyperlipidemia-induced inhibition of insulin signaling in both differentiated C2C12 cells and mouse skeletal muscle [12]. In the current study, we report for the first time that BAIBA treatment significantly increased insulin-stimulated IRS-1 and Akt phosphorylation, and glucose uptake in mouse adipocytes, suggesting that BAIBA has an anti-diabetic effect.

Jung et al. have documented that BAIBA exerts its anti-obesity effect by stimulating fatty acid oxidation in skeletal muscle [12]. In the current study, we found that BAIBA treatment reduced lipogenic-genes (*Fabp4, Pparγ, adiponectin, FAS*) mRNA expression accompanying a decrease in lipid accumulation during differentiation of 3 T3-L1 cells. Furthermore, BAIBA stimulated fatty acid oxidation. These results suggest that BAIBA has potential anti-obesity effects in adipocytes. Furthermore, these results support data describing body weight loss by BAIBA administration in a previous study [12]. However, further investigation is required to elucidate detailed mechanisms for suppression of adipocyte differentiation by BAIBA.

Glimepiride, a third-generation diabetes drug sulfonylurea, has demonstrated not to have any effects on AMPK [33]. In this study, we demonstrated that glimepiride attenuated LPS-induced pro-inflammatory cytokines secretion and glucose uptake impairment, but it was less effective than BAIBA (Additional file 3: Figure S3). Therefore, AMPK plays a crucial role in the beneficial effects of BAIBA on inflammation and insulin resistance in differentiated adipocytes. We next compared the effects of BAIBA on inflammation and insulin resistance with metformin, an anti-diabetic drug via AMPK activation [34]. Metformin demonstrated the strongest suppressive effects. But, BAIBA is less effective than metformin (Additional file 3: Figure S3). These results demonstrate that metformin is the most effective for suppression of inflammation and insulin resistance. However, side effects such as hypoglycemia (sulfonylurea; [35]), weight gain (sulfonylurea; [35]) or hepatotoxicity (metformin; [36]), may be less, because BAIBA is an endogenous substance. In addition, doses of BAIBA are much less than metformin. Therefore, BAIBA seems to be worth developing as a safer drug for treatment of insulin resistance at least from the perspective of adipocytes.

In summary, we investigated the effects of BAIBA on fully differentiated 3 T3-L1 cells to elucidate whether BAIBA could ameliorate inflammatory responses in adipocytes and subsequently obesity-induced low-grade chronic inflammation in adipose tissue. Our current findings verify that BAIBA alleviates LPS-induced inflammatory responses via AMPK-associated suppression of NFκB-dependent signaling. Not only did BAIBA prevent lipid accumulation during differentiation of 3 T3-L1 cells, but it also ameliorated insulin resistance in differentiated 3 T3-L1 cells.

## Conclusion

**Fig. 5:**
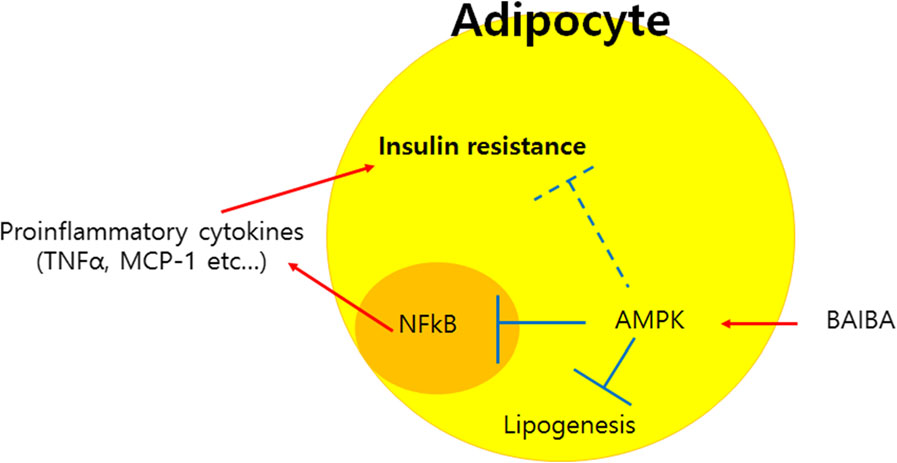
Schematic diagram of the effects of BAIBA on inflammation and insulin resistance in adipocytes. BAIBA administration stimulates AMPK activation, resulting in suppression of lipogenesis, inflammation, and insulin resistance in differentiated adipocytes

The present study found that BAIBA ameliorates LPS-induced pro-inflammatory responses and insulin resistance in differentiated adipocytes through AMPK-mediated signaling. Furthermore, BAIBA markedly suppresses lipid accumulation during 3 T3-L1 cell differentiation and stimulates fatty acid oxidation via AMPK pathway (Fig. 5). Attenuation of pro-inflammatory cytokines such as IL-6, TNFα, and IL-1β secreted by immune cells [27, 37] and adipocytes [38, 39] may be a therapeutic approach for treating systemic low-grade chronic inflammation-associated with metabolic syndrome. Therefore, our report may suggest a novel strategy for obesity-associated metabolic disorders including insulin resistance.

## Additional files

Additional file 1: **Figure S1**. Selection of optimized concentrations of glimepiride and metformin for cell treatment. Cell viability measured by MTT assay in differentiated 3 T3-L1 cells treated with (a) BAIBA (0–30 μM), (b) glimepiride (0–5 μM), or (c) metformin (0–20 mM) for 10 days. Culture media analysis of TNFα and MCP-1 in differentiated 3 T3-L1 cells treated with 10 μg/ml LPS for 24 h and glimepiride (0–5 μM) (d) or metformin (0–200 μM) (e) for 10 days. Means ± SEM were calculated data obtained from three independent experiments. ^***^P < 0.001 and ^**^P < 0.01 when compared to the control.^!!!^*P* < 0.001,^!!^*P* < 0.01, and^!^*P* < 0.05 when compared to the LPS treatment (TIFF 4230 kb)

Additional file 2: **Figure S2**. Selection of optimized concentrations of glimepiride and metformin for cell treatment. Cell viability measured by MTT assay in differentiated 3 T3-L1 cells treated with (a) BAIBA (0–30 μM), (b) glimepiride (0–5 μM), or (c) metformin (0–20 mM) for 24 h. Culture media analysis of TNFα and MCP-1 in differentiated 3 T3-L1 cells treated with 10 μg/ml LPS for 24 h and glimepiride (0–5 μM) (d) or metformin (0–20 mM) (e) for 24 h. Means ± SEM were calculated data obtained from three independent experiments. ^***^P < 0.001 when compared to the control.^!!!^*P* < 0.001,^!!^*P* < 0.01, and^!^*P* < 0.05 when compared to the LPS treatment (TIFF 4230 kb)

Additional file 3: **Figure S3**. Comparison of BAIBA, metformin, and glimepiride effects on inflammation and insulin resistance. (a) Culture media analysis of TNFα and MCP-1 and (b) 2-deoxyglucose uptake in differentiated 3 T3-L1 cells treated with 10 μg/ml LPS for 24 h and BAIBA (30 μM), metformin (0.1 mM) or glimepiride (5 μM) for 10 days. (c) Culture media analysis of TNFα and MCP-1 and (d) 2-deoxyglucose uptake in differentiated 3 T3-L1 cells treated with 10 μg/ml LPS and BAIBA (30 μM), metformin (10 mM) or glimepiride (5 μM) for 24 h. Human Insulin (10 nM) stimulates glucose uptake for 30 min. Means ± SEM were calculated data obtained from three independent experiments. ^***^P < 0.001 when compared to the control or insulin treatment.^!!!^*P* < 0.001,^!!^*P* < 0.01, and^!^*P* < 0.05 when compared to the LPS treatment or insulin plus LPS treatment. ^###^*P* < 0.001, ^##^*P* < 0.01, and ^#^*P* < 0.05 when compared to the LPS plus BAIBA treatment (TIFF 3804 kb)

## Abbreviations

AMPK: AMP-activated protein kinase
BAIBA: β-aminoisobutyric acid
LPS: Lipopolysaccharide
NFκB: Nuclear factor kappa beta
siRNA: Small interfering RNA
