# Supplementary figures and images for: β-aminoisobutyric acid attenuates LPS-induced inflammation and insulin resistance in adipocytes through AMPK-mediated pathway
Source: J Biomed Sci. 2018 Mar 28;25:27. doi: 10.1186/s12929-018-0431-7 (PMC5875012; doi:10.1186/s12929-018-0431-7)

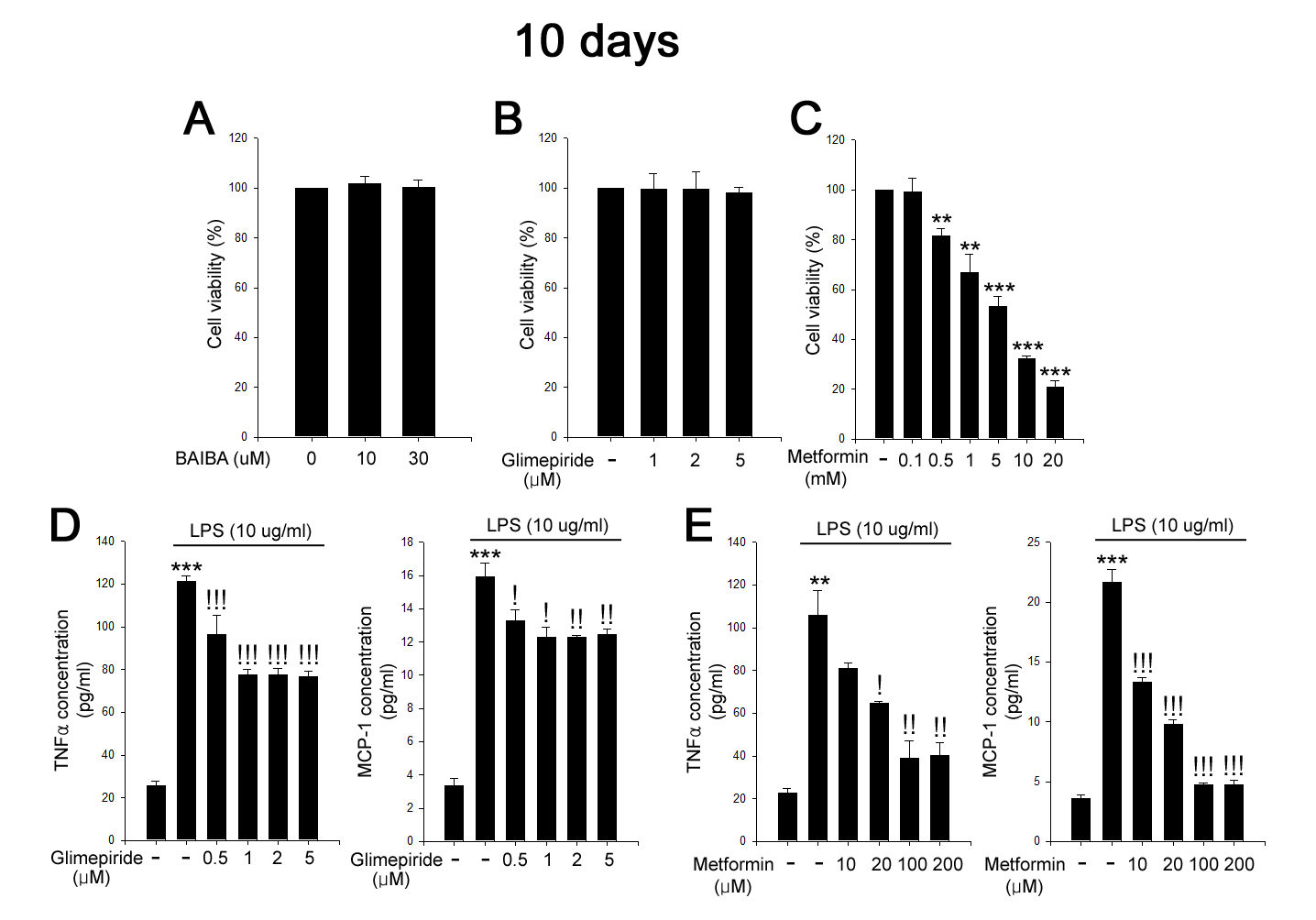

Supplement: Supplementary file 1 — Figure S1. Selection of optimized concentrations of glimepiride and metformin for cell treatment. Cell viability measured by MTT assay in differentiated 3 T3-L1 cells treated with (a) BAIBA (0–30 μM), (b) glimepiride (0–5 μM), or (c) metformin (0–20 mM) for 10 days. Culture media analysis of TNFα and MCP-1 in differentiated 3 T3-L1 cells treated with 10 μg/ml LPS for 24 h and glimepiride (0–5 μM) (d) or metformin (0–200 μM) (e) for 10 days. Means ± SEM were calculated data obtained from three independent experiments. ***P < 0.001 and **P < 0.01 when compared to the control.!!!P < 0.001,!!P < 0.01, and!P < 0.05 when compared to the LPS treatment (TIFF 4230 kb) [file 12929_2018_431_MOESM1_ESM.tif]

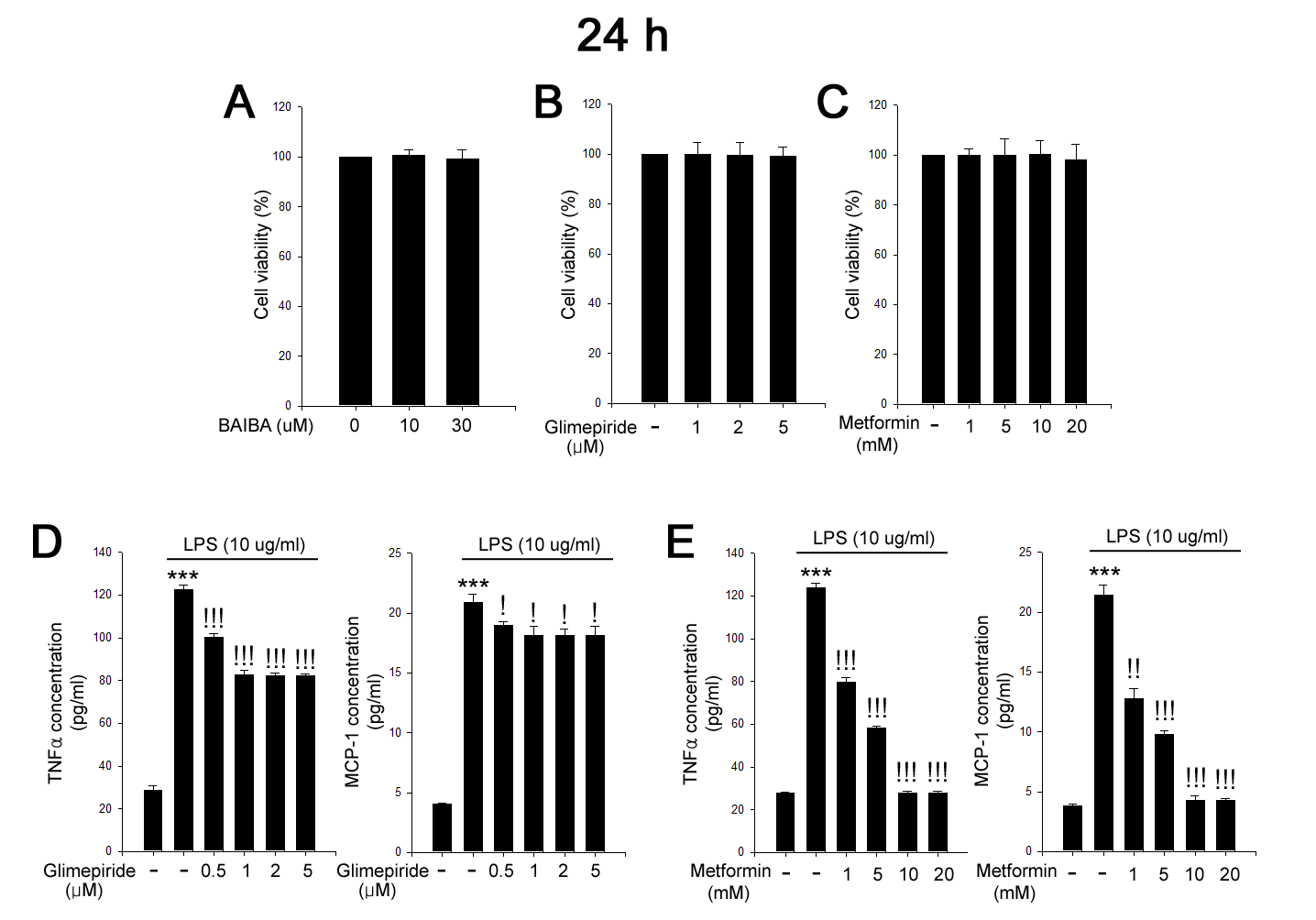

Supplement: Supplementary file 2 — Figure S2. Selection of optimized concentrations of glimepiride and metformin for cell treatment. Cell viability measured by MTT assay in differentiated 3 T3-L1 cells treated with (a) BAIBA (0–30 μM), (b) glimepiride (0–5 μM), or (c) metformin (0–20 mM) for 24 h. Culture media analysis of TNFα and MCP-1 in differentiated 3 T3-L1 cells treated with 10 μg/ml LPS for 24 h and glimepiride (0–5 μM) (d) or metformin (0–20 mM) (e) for 24 h. Means ± SEM were calculated data obtained from three independent experiments. ***P < 0.001 when compared to the control.!!!P < 0.001,!!P < 0.01, and!P < 0.05 when compared to the LPS treatment (TIFF 4230 kb) [file 12929_2018_431_MOESM2_ESM.tif]

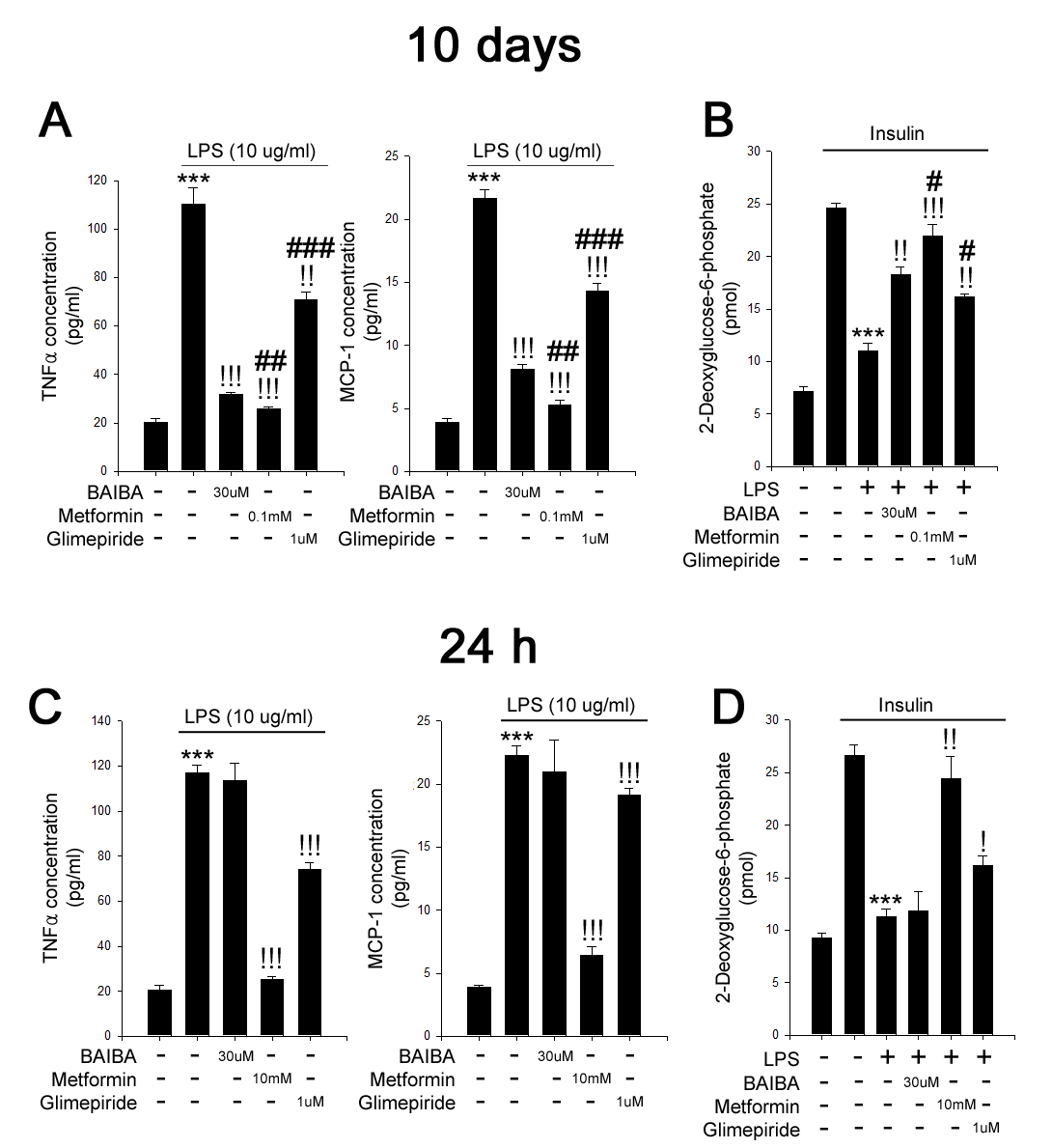

Supplement: Supplementary file 3 — Figure S3. Comparison of BAIBA, metformin, and glimepiride effects on inflammation and insulin resistance. (a) Culture media analysis of TNFα and MCP-1 and (b) 2-deoxyglucose uptake in differentiated 3 T3-L1 cells treated with 10 μg/ml LPS for 24 h and BAIBA (30 μM), metformin (0.1 mM) or glimepiride (5 μM) for 10 days. (c) Culture media analysis of TNFα and MCP-1 and (d) 2-deoxyglucose uptake in differentiated 3 T3-L1 cells treated with 10 μg/ml LPS and BAIBA (30 μM), metformin (10 mM) or glimepiride (5 μM) for 24 h. Human Insulin (10 nM) stimulates glucose uptake for 30 min. Means ± SEM were calculated data obtained from three independent experiments. ***P < 0.001 when compared to the control or insulin treatment.!!!P < 0.001,!!P < 0.01, and!P < 0.05 when compared to the LPS treatment or insulin plus LPS treatment. ###P < 0.001, ##P < 0.01, and #P < 0.05 when compared to the LPS plus BAIBA treatment (TIFF 3804 kb) [file 12929_2018_431_MOESM3_ESM.tif]
